# The case to scale up edutainment as an effective public health communication intervention to combat the COVID-19 pandemic in Zimbabwe

**DOI:** 10.34172/hpp.2022.05

**Published:** 2022-05-29

**Authors:** Tafadzwa Dzinamarira, Brian Nachipo, Albert Nyathi, Roda Madziva, Helena Herrera, Hugh Siegel, Godfrey Musuka

**Affiliations:** ^1^School of Health Systems & Public Health, University of Pretoria, Pretoria, 0002, South Africa; ^2^ICAP at Columbia University, Harare, Zimbabwe; ^3^Ministry of Health and Child Care, AIDS and TB Control Programme, Harare, Zimbabwe; ^4^Independent Consultant, Harare, Zimbabwe; ^5^School of Sociology and Social Policy, University of Nottingham, Nottingham, UK; ^6^School of Pharmacy and Biomedical Sciences, University of Portsmouth, Portsmouth, UK; ^7^ICAP at Columbia University, New York, USA

**Keywords:** COVID-19, Public health, Communication

## Abstract

Efforts to control the coronavirus disease 2019 (COVID-19) pandemic have been negatively affected by myths, misconceptions and misinformation, fuelled by an "infodemic" spread via social media platforms. In response, The Zimbabwean COVID-19 response built on its experience with past public health communication strategies to employ edutainment strategies for COVID-19 awareness campaigns. This article discusses the different strategies and how they were employed. In this perspective piece, the authors discuss edutainment as an effective vehicle for reaching wider sectors of society. In tackling complex social issues with simple language, integrated into various entertainment formats, edutainment can bring about change in contexts where traditional strategies and actions may prove unsuccessful.

## Introduction

Coronavirus disease 2019 (COVID-19) became a major public health problem characterized by high infectivity of the severe acute respiratory syndrome coronavirus 2 (SARS-CoV-2), and high morbidity and fatality rates. The pandemic that it caused introduced a “new normal,” where restrictions to movement were aimed at slowing down the spread of this respiratory infection. The high infectivity and mortality rates, along with the restrictions, have had a significant psychosocial impact, causing mental and emotional health problems, substantial economic burden and financial losses.^1,2^ Social distancing and movement restrictions disrupted interpersonal and community interactions, severely impacting the social fabric globally.^3^

Efforts to control this pandemic have been negatively affected by myths, misconceptions and misinformation, fuelled by an “infodemic” spread via social media platforms^4^ and other communications channels.^5,6^ While this approach was an important step in COVID-19 health communication, it may have limited impact due to the very nature of the problems it was aiming to address: overexposure to conflicting information alongside distrust of the government. Recognizing this, the country built on its experience with HIV and AIDS, malaria, and tuberculosis health communication strategies to mobilize a team of health promotion, communications, and technical experts to lead additional COVID-19 awareness campaigns. As a result, “edutainment” emerged as a key strategy to focus COVID-19 public health information dissemination efforts.

## Edutainment as a response to public health emergencies

Edutainment is defined as the purposeful use of entertainment media to educate viewers about specific issues of a target population.^7^ In this regard,edutainment provides an opportunity to educate audiences using approaches that go beyond the traditional didactic methods, providing information in an accessible and entertaining way designed both to create awareness and influence behaviour change. Edutainment is grounded in the social learning theory,^7,8^ which posits that observing, modelling, and imitating the behaviors, attitudes, and emotional reactions of others can influence human learning and behavior.^9^ Music, roadshows, television dramas, community dramas, comics and animations, radio and film can all become forms of edutainment when infused with informational messaging.^10^

Edutainment strategies in Zimbabwe have successfully addressed public health threats such as malaria, tuberculosis, and HIV/AIDS over the years. An example of successful use of this approach would be the use of local musicians to support the male circumcision programme.^11^ Another example is the mobilization of the population to participate in the PEPFAR-supported Population-Based HIV Impact Assessment (PHIA) surveys in Zimbabwe also by using the “knock-knock song”,^12^ played widely over the radio and television. It is against this background that a Risk Communication and Community Engagement (RCCE) strategy was developed by the Ministry of Health and Child Care,^13^ using a combination of edutainment strategies to bring public awareness of COVID-19. This article discusses the different strategies and how they were employed.

## Use of edutainment in combating COVID-19 in Zimbabwe

The Zimbabwe Ministry of Health and Child Care introduced several edutainment strategies, including roadshows, music, jingles, poetry, and community drama, to communicate COVID-19 related health messages.

### COVID-19 roadshows 

A roadshow is a promotional presentation, typically conducted in a series of locations.^14^ In Zimbabwe, roadshows generally employ a branded van or truck with trained personnel who educate communities as the truck circulates to different venues. A specific example of this is the ‘*in-your-face’* nationwide campaign, which was conducted in March 2020 to address low COVID-19 risk perception, myths and misconceptions, and spread information on mask wearing, social distancing, and hand washing. During these shows, the communities were educated through skits and song performances. Within the first month of implementation, the campaign had reached more than half a million people in urban and rural areas of Zimbabwe.^15^

### COVID-19 music, jingles, and poetry

In Zimbabwe, a number of stakeholders stepped up to promote the use of music as a weapon in the battle against the COVID-19 pandemic. A few weeks into the COVID-19 outbreak, ICAP at Columbia University, a global health center that has been supporting the HIV response in the country for several years, commissioned the COVID-19 anthem “Apart/Together – We Stand Strong Against COVID-19.” This song blended English, Shona and Ndebele to celebrate community efforts to fight the pandemic, while also providing health protection information against COVID-19.^16^ Another initiative that used music as edutainment was sponsored by the United Nations in Zimbabwe, which held virtual concerts shared online via social media. These concerts, which featured nine top Zimbabwean musicians performing songs to disseminate preventative measures and highlight society’s role in curbing the spread of the disease reached over one million people.^17^

The Ministry of Health and Child Care also employed jingles and poems to communicate technical and medical terms related to COVID-19 in simple language, blended with entertainment, to make the message more accessible to the audience. For example, a jingle titled ‘Corona,’ targeted the minority Tsonga people using their own dialect.^18^ A teenager, Elvis Mazwimairi, was inspirational in communicating COVID-19 awareness to the youth with his poem, “I am Corona”.^19^

### COVID-19 dramas and puppetry

The COVID-19 health communication in Zimbabwe also employed interactive media formats such as dramas and puppetry. These were employed particularly to reach children. While children often prove difficult to reach with public health messages, UNICEF has reported that children have shown a positive response to puppet shows with COVID-19 messages incorporated.^20^One fifth-grade student was quoted as saying: “*I want more of such educative dramas. I hope (Tutu) returns next time so my friends who are not here can also learn. We are going back to school soon and I learnt a lot about how to stay safe from COVID-19*.”^20^

These and other such initiatives that have helped to reach different groups of people in society demonstrate that edutainment can be an important strategy in the fight against the COVID-19 pandemic.

## The case to scale up edutainment as part of the COVID-19 response in Zimbabwe

Since COVID-19 is an emerging disease, the experience of managing the crisis it has provoked, and engaging with communities to respond has been an ongoing process with many lessons along the way. One such lesson in Zimbabwe has been that edutainment is a powerful tool to influence adoption or change of behaviour, particularly in hard-to-reach populations. Furthermore, edutainment can tackle deeply rooted social norms, such as superstitions, distrust and reluctance to comply with COVID-19 restrictions and measures.

While the impact of edutainment specifically in the Zimbabwean COVID-19 response is still to be evaluated, there is ample empirical evidence that edutainment is an effective tool for overcoming communication barriers in a public health crisis. For instance, one of the advantages of edutainment is that it can reach populations with low levels of literacy. Given the promising outcomes we have seen to date, it follows that more should be done to strengthen this approach. We suggest that segmentation should take place to dig deeper into various groups’ psychological profiles, enabling targeted messaging and interventions for each population segment.^21^ Doing this would help to ensure that interventions are targeted toward the key barriers to compliance with recommended behaviours, allowing interventions to focus on key motivators to desired behaviours.

With the availability of vaccines, the COVID-19 pandemic response in Zimbabwe now includes the need for widespread vaccination to curb spread and reduce morbidity and mortality. A rapid national survey, conducted in February 2021, revealed 50% of the population were hesitant to get vaccinated.^22^ Another study conducted in May 2021 revealed that reluctance to get vaccinated was most strongly associated with lack of confidence in vaccine safety.^23^ Edutainment interventions may be useful in segmentation process in order to identify the key issues within each population segment to enable the designing of tailored messaging that help build public confidence on vaccine safety, driving uptake. At the time of writing this article, edutainment is still not in use for promoting COVID-19 vaccination in Zimbabwe.

## Conclusion

Edutainment can be an effective vehicle for reaching wider sectors of society, and, as it can be tailored towards specific groups, particularly for reaching underserved communities and groups more reluctant to modify behaviours. In tackling complex social issues with simple language, integrated into various entertainment formats, edutainment can bring about change in contexts where traditional strategies and actions may prove unsuccessful.

## Authors’ contributions

TD and BN prepared the first draft of the paper and subsequent reviews and revisions were completed by all authors. All authors reviewed the final draft and gave approval to publish.

## Funding

This research received no external funding

## Ethical approval

Not applicable.

## Competing interests

The authors declare that they have no competing interests.
